# Frontiers in aptamer-based diagnostics and therapeutics for zoonotic diseases

**DOI:** 10.7717/peerj.21304

**Published:** 2026-05-29

**Authors:** Jia Ying Wong, Mai Abdel Haleem A. Abusalah, Nurul Izzaty Najwa Zahari, Wardah Yusof, Lih Huey Lee, Choo Yee Yu, Geik Yong Ang, Abdul Rahman Zaidah, Chan Yean Yean

**Affiliations:** 1 Department of Medical Microbiology and Parasitology, School of Medical Sciences, Universiti Sains Malaysia, Kubang Kerian, Kelantan, Malaysia; 2 Department of Medical Laboratory Sciences, Faculty of Allied Medical Sciences, Al-Ahliyya Amman University, Amman, Jordan; 3 Laboratory of Vaccine and Biomolecules, Institute of Bioscience, Universiti Putra Malaysia, Serdang, Selangor, Malaysia; 4 Faculty of Sports Science and Recreation, Universiti Teknologi MARA, Shah Alam, Selangor, Malaysia

**Keywords:** Aptamer, Zoonotic disease, Biosensor, Diagnostics, Theranostics, SELEX, Therapeutics

## Abstract

Zoonotic diseases have become a significant public health concern due to their capacity to cross species barriers and cause widespread outbreaks. Conventional diagnostic and therapeutic strategies often lack the specificity, sensitivity, and rapid adaptability required for effective disease management. Therefore, aptamers, single-stranded oligonucleotides, have emerged as powerful tools in detection and therapeutics. This review elucidates the role of aptamer-based technologies in diagnosing and treating bacterial, viral, and parasitic zoonoses, including *Leptospira*, *Helicobacter pylori*, rabies virus, monkeypox virus, *Plasmodium*, and *Toxoplasma* *gondii*. It highlights their mechanisms of interaction, including the molecular recognition of virulence factors, such as lipoprotein L32 (*lipL32*), non-structural protein 1 (NS1), and *Plasmodium falciparum* lactate dehydrogenase (PfLDH). It explores the development of aptamer-based biosensors, theranostic systems, and point-of-care (POC) detection platforms. Furthermore, emerging innovations such as nanomaterial conjugation, artificial intelligence-assisted systematic evolution of ligands by exponential enrichment (SELEX), and microfluidic integration are discussed as transformative approaches that enhance diagnostic performance and field applicability. Collectively, this review underscores the versatility and clinical promise of aptamers as next-generation biomolecular tools for rapid, accurate, and cost-effective management of zoonotic diseases, addressing the limitations of existing diagnostic and therapeutic modalities.

## Introduction

Zoonotic diseases are infections transmitted between humans and animals, representing more than 60% of all infectious diseases worldwide (World Health Organization (WHO), 2023). These diseases, caused by bacteria, viruses, fungi, or protozoa, spread through direct animal contact, contaminated food or water, aerosolised droplets, or infected vectors (Vourc’H et al., 2022; Tumelty et al., 2023; Elsohaby & Villa, 2023). Their frequent outbreaks pose major public health and economic burdens, demanding substantial healthcare resources for treatment and prevention (Elsohaby & Villa, 2023). Diagnosis and treatment remain challenging due to overlapping symptoms, unidentified sources of infection, and limited diagnostic tools (Humayun et al., 2023). Misdiagnosis can delay treatment and lead to severe outcomes, while antimicrobial resistance and poor coordination between human and veterinary sectors further complicate disease management (World Health Organization (WHO), 2020). These issues highlight the need for rapid, sensitive, and cost-effective diagnostic and therapeutic platforms.

Aptamers have emerged as promising biorecognition elements to address these challenges. They are short, single-stranded DNA or RNA molecules that fold into unique three-dimensional structures capable of highly specific, high-affinity binding to their targets (Tuerk & Gold, 1990). Compared with antibodies, aptamers exhibit superior thermal stability, cost-effectiveness, and ease of modification (Thiviyanathan & Gorenstein, 2012; Liu et al., 2021). Building on these advantages, aptamer-based biosensors (aptasensors) have been successfully applied in food safety, environmental monitoring, and clinical diagnostics, demonstrating excellent sensitivity, specificity, and portability (Li et al., 2019; Phopin & Tantimongcolwat, 2020; Prante et al., 2020).

**Figure 1:**
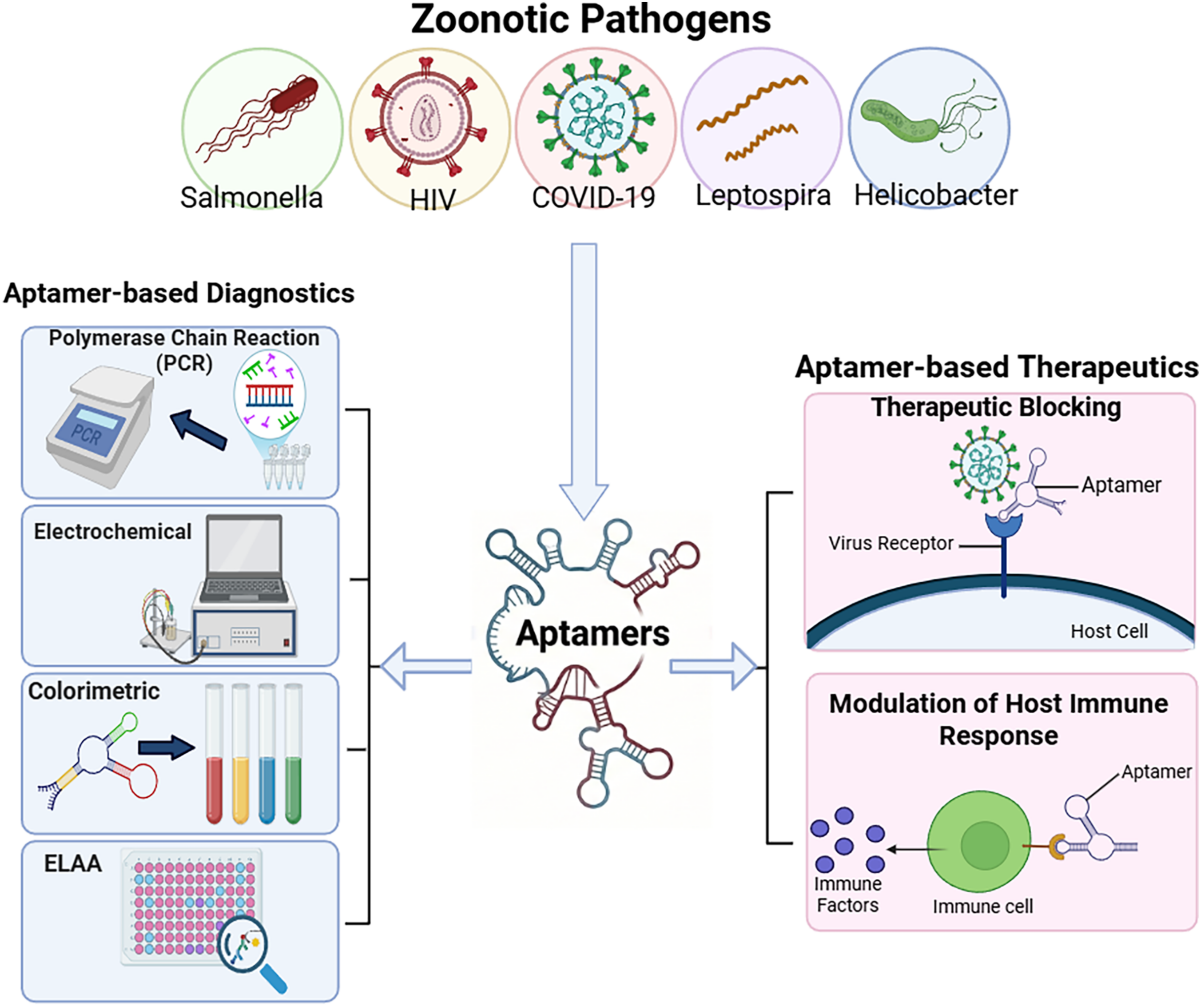
Diagnostic and therapeutic applications of aptamers against zoonotic pathogens. Aptamers can be engineered to target specific zoonotic agents (top). These molecules are utilized in diverse diagnostic platforms (left), including PCR, electrochemical, colorimetric, and ELAA systems, and serve as therapeutic agents (right) by blocking pathogen entry or modulating host immune factors. Figure created in Biorender (https://BioRender.com).

For zoonotic diseases, aptasensors enable rapid near-patient testing across multiple readout formats, support minutes-to-result workflows, and can be used with minimally processed samples. Table 1 summarises the developed diagnostic aptamers and their targets, highlighting architectures that maximise analytical performance while maintaining point-of-care (POC) feasibility. Beyond diagnostics, aptamers are emerging as therapeutic agents. They can neutralise toxins, block pathogen adhesion or entry, and modulate host immune responses, either as standalone molecules or as part of targeted delivery systems (Shraim et al., 2022; Ye et al., 2024; Sujith et al., 2024). Table 2 outlines current aptamers developed for therapeutic applications against zoonotic pathogens, while Fig. 1 illustrates representative roles of aptamers across diagnostic and therapeutic pipelines.

**Table 1:** Summary of aptamers developed in recent years in diagnostic application of zoonotic diseases.

Microorganisms	Diseases	Species	Target protein	Aptamer name	Dissociation constant (Kd)	Limit of Detection (LOD)	Detection platform	References
Bacteria	Leptospirosis	*Leptospira interrogans*		N/A	356.6 nM	119 CFU/mL	DNAzyme-based colorimetric sensor	Saranya et al. (2024)
			ETFB protein	ETFB3-63	50–500 nM	N/A	Electrochemical aptasensor	Kositanont et al. (2023)
			*lip* *L32*	LepRapt-11	350 ± 47.45 nM	100 nM	ELASA	Shien Yeoh et al. (2023)
			*lip* *L32*	LAP3	133.13 nM	57 CFU/mL	Colorimetric aptamer-AuNP assay	Vishwakarma, Meganathan & Ramya (2023)
			*lip* *L32*	LepDapt-5a	33.97 ± 5.30 nM–46.35 ± 9.09 nM	10^5^ CFU/mL	Sandwich ELASA	Yeoh, Tang & Citartan (2023)
						10^6^ CFU/mL	Direct ELASA	
						10^3^ CFU/mL	Dot-blot	
	Helicobacter	*Helicobacter pylori*		Hp4	26.48 ± 5.72 nmol/L	N/A	NA	Yan et al. (2019)
				HPA-2	19.3 ± 3.2 nM	88 CFU/mL	Fluorescense microscope	Wu et al. (2021)
			Cytotoxin-associated gene A (Cag A)	N/A	0.1–160 ng/mL	0.017 ng/mL	Electrochemical aptasensor	Yadav et al. (2023)
				N/A	N/A	33 CFU/mL	Molecularly imprinted (MIP)-aptasensor	Roushani, Sarabaegi & Hosseini (2024)
				AptHlyE11	102.2 nM	N/A		
				AptHlyE45	119.3 nM	N/A		
Virus	Monkey pox		A29 protein of MPXV	A29-Apt41	6.8 pM	0.28 ng/mL	CRISPR/Cas12a-mediated aptasensor	Han et al. (2024)
				A29-Apt12	56.4 pM			
				MANGO III		Four copies	Recombinase-aided amplification (RAA)-CRISPR-Cas13a-Apt assay	Wang et al. (2024)
	ZIKA	Zika virus (ZIKV)	Non-structural protein 1 (NS1)	ZIKV60	2.28 nM	0.01 pg/mL	Electrochemical Aptasensor—differential pulse voltammetry (DPV)	Almeida et al. (2022)
			Zika virus envelope protein	truncated ZV aptamer (T-ZV apt)	333.1 nM	90.1 pM	Electrochemical Aptasensor—alternating current electrothermal flow (ACEF)	Jang et al. (2023)
			Envelope protein (ZIKV Env)	B2.33 (capture peptide) and P6.1 (detector peptide)	N/A	2 × 10^4^ TCID50/mL	Peptide pair-based FICT assay	Nguyen et al. (2022)
Parasite	Malaria	*Plasmodium vivax*	Plasmodium lactate dehydrogenase (LDH)	pL1	N/A	22.3 fM (50 parasites/µL)	Electrochemical multielectrode array	Figueroa-Miranda et al. (2021)
		*Plasmodium falciparum*	*Plasmodium falciparum* histidine rich protein 2 (PfHRP2)	2106s	29.53 nM	3.73 nM	Electrochemical aptamer-based sensor	Lo et al. (2021)
			Plasmodium lactate dehydrogenase (LDH)	LDHp11	N/A	1.80 fM (<50 parasites/µL)	Electrochemical multielectrode array	Figueroa-Miranda et al. (2021)
			Plasmodium lactate dehydrogenase (PfLDH)	2008s	2.4 nM	0.78 fM (0.11 pg/mL)	Graphene-based 2DBioFET aptasensor	Figueroa-Miranda et al. (2022)
			Glutamate dehydrogenase (PfGDH)	NG3	N/A	264 pM	Smartphone-based fiber-optic aptasensor	Sanjay et al. (2020)
	Toxoplasmosis	*Toxoplasma gondii*	Surface antigen 1 (SAG1)	aptamer-2	41.57 pM	N/A	Direct enzyme-linked aptamer assay (DELAA)	Cui et al. (2022)
			Rhoptry protein 18 (ROP18)	AP001	62.7 ± 17.27 nM	1.56 μg/mL	Direct enzyme-linked aptamer assay (DELAA)	Vargas-Montes et al. (2019)
						3.12 μg/mL	Sandwich enzyme-linked aptamer assay (ELAA)	
				AP002	97.7 ± 22.20 nM	N/A	Direct enzyme-linked aptamer assay (DELAA)	
			Antitoxoplasma IgG	TGA6 & TGA7	N/A	0.1 IU	Quantum dots-labeled dual aptasensor (Q-DAS)	Luo et al. (2013)

**Table 2:** List of aptamers developed in recent years in therapeutic application of zoonotic diseases.

Microorganisms	Diseases	Species	Target protein	Aptamer name	Function	References
Bacteria	Leptospirosis	N/A	*lip* *L32*	N/A	Stops the bacteria invasion activity and control the interactions between *lipL32* and ECM components	Hsu et al. (2024)
	Helicobacter	N/A	HpaA	HA6	Effectively disrupted the pathway and reduced IL-21 levels and therefore inhibited metastasis	Xia et al. (2020)
		N/A	Blood group antigen-binding adhesin (BabA)	A10, A41, A42, A3 and A16	Effectively inhibit the bacteria’s adherence *in vitro*, reduce the inflammatory responses and aids in prevention of bacteria colonization on the mice stomach mucosa	Yuan et al. (2023)
Virus	Rabies	N/A	CVS-11	FO21 and FO24	Stop replication of standard stranded rabies virus (RABV), CVS-11, with high survival rate of 87.5%	Liang et al. (2013)
		N/A	N/A	GE54	Offered partial protection even 48 h post-infection giving rise to survival rates between 25–33%	Liang et al. (2014)
		N/A	Rabies virus glycopeptide (RVG)	N/A	A 100× reduction in viral load in cell supernatants and at the same time lowered viral RNA levels in mouse brains post-PTT. The therapy resulted in a 60% survival rate among infected mice with minimal side effects which include no notable inflammation or apoptosis to brain tissues	Ren et al. (2021)
		N/A	N/A	UPRET 2.03	Explored the potential of aptamers as a post-exposure prophylaxis (PEP) alternative to rabies immunoglobulin (RIG), reduced viral RNA by 61.3% at 2 h post-infection	Scott & Nel (2021)
Parasite	Malaria	*Plasmodium falciparum*	tRip membrane protein	Aptamer-15	Aptamers compete with tRNAs for binding to tRip, suggesting that they can block tRip function and slow parasite development	Pitolli et al. (2024)
		*Plasmodium falciparum*	CD36	RC60 and RC25	As anticytoadherence for severe malaria adjunct therapy	Nik Kamarudin et al. (2020)

Although several reviews have examined aptamer-based diagnostics and therapeutics, most focus on specific disease classes or individual technological platforms. In contrast, this review centres on zoonotic diseases and integrates bacterial, viral, and parasitic pathogens within a unified framework. Emphasis is placed on emerging themes such as advances in aptamer selection strategies (including SELEX), biosensor integration, nanomaterial conjugation, and artificial intelligence-assisted aptamer design, by critically summarising current aptamer-based approaches for zoonotic diagnostics and therapeutics. This review aims to identify knowledge gaps, assess translational readiness, and highlight key challenges and opportunities for future clinical and field implementation. This work is intended for an interdisciplinary audience, including microbiologists, molecular biotechnologists, and diagnostic innovators, seeking an integrated overview of how aptamer-based systems may advance the detection and treatment of zoonotic diseases.

## Survey methodology

A structured narrative literature survey was conducted to identify and synthesise studies reporting aptamer-based diagnostics and/or therapeutics for zoonotic pathogens. Searches were performed in PubMed, Web of Science, Scopus and Google Scholar for publications from 2010 to 2025 using combinations of the following keywords including: aptamer, SELEX, aptasensor, diagnostics, therapeutics, theranostics, zoonotic disease, bacteria, virus, parasite, artificial intelligence, and microfluidic, along with pathogen-specific terms (*Leptospira, Helicobacter pylori*, rabies virus, monkeypox virus, *Plasmodium, Toxoplasma gondii*). In addition, guidance documents and situation reports from authoritative agencies (WHO and CDC) were consulted to contextualise the relevance of zoonotic disease and translational needs.

Selected articles underwent systematic screening and categorisation by pathogen type and diagnostic or therapeutic role. Key data, including aptamer, target proteins, dissociation constants (K_d_), detection platforms, and analytical performance metrics, were extracted and compiled in Table 1 (diagnostic applications) and Table 2 (therapeutic applications). In addition, Fig. 1 illustrates the aptamer-based framework for zoonotic disease control. Findings were synthesised to highlight trends, strengths and limitations, and future trajectories in aptamer technology, offering critical insights into their translational viability for real-world diagnostic and therapeutic use. The imbalance of literature across pathogens or technologies was attributed to publication availability, with potential bias from under-represented pathogens acknowledged in the discussion.

### Aptamer applications for bacterial zoonotic pathogens

Bacterial zoonoses can spread through direct contact with infected animals, consumption of pathogen-contaminated food or water, inhalation of aerosols, or through vectors like fleas and ticks. To date, common bacterial zoonotic diseases posing significant public health concerns worldwide include *helicobacter* infection and leptospirosis (González-Barrio, 2022).

### Helicobacter

Gastritis and peptic ulcers are digestive diseases caused by the helical-shaped, gram-negative zoonotic bacterium, *Helicobacter pylori*. This bacterium has been detected in companion pets, mainly in dogs and cats (Moussa et al., 2021). Globally, about 4.4 billion humans have been infected through faecal-oral or oral-oral transmission (Martínez et al., 2019; Bashir & Khan, 2023; Duan et al., 2023). Infection with *H. pylori* is strongly associated with gastric mucosal lymphoid tissue lymphoma, duodenal ulcers, carcinomas, stomach cancer, and peptic ulcer disease (Elbehiry et al., 2023).

The current diagnostic method for *H. pylori* is divided into invasive and non-invasive techniques (Ali & Alhussaini, 2024). Non-invasive techniques include serological assays, stool antigen tests (SAT) and urea breath tests (UBT). Serological tests are accurate for initial screening but unreliable for eradication confirmation due to persistent antibodies after treatment (Malfertheiner et al., 2012). UBT detects urease activity produced by *H. pylori*, which is highly specific and sensitive. However, it may cause false positives due to urease activity from other gut bacteria (Patel et al., 2014). Similarly, the SAT provides reliable detection, but it may be less effective in patients with low bacterial loads or after antibiotic use. Invasive techniques, such as endoscopic biopsy followed by histology, culture, or polymerase chain reaction (PCR), offer higher diagnostic certainty but are limited by invasiveness, cost, and technical complexity (Patel et al., 2014). These limitations highlight the need for advanced diagnostic tools for a faster, more accurate, and less invasive detection of *H. pylori*.

In response to these challenges, aptamer-based approaches have emerged as promising tools for *H. pylori* detection. Yan et al. (2019) developed DNA aptamers targeting the bacterial surface recombinant antigen (HP-Ag), among which FAM-labelled aptamer Hp4 exhibited the highest affinity (K_d_ = 26.48 ± 5.72 nmol/L). Cross-reactivity studies against *V. anguillarum, S. aureus* and *E. coli* demonstrated strong specificity for *H. pylori*, supporting its use in selective detection. Similarly, Wu et al. (2021) developed a portable torchlight sensor using the aptamer HPA-2 (K_d_ = 19.3 ± 3.2 nM) as the recognition element, enabling naked-eye detection of *H. pylori* within 5 min. Compared to conventional assays, these platforms provide faster responses and simpler operation, although validations were conducted primarily with food or laboratory-prepared samples instead of clinical specimens.

Integrating aptamers into advanced platforms such as electrochemical sensors has further enhanced the specificity and sensitivity of *H. pylori* detection. Yadav et al. (2023) developed an electrochemical aptasensor targeting the virulence factor CagA of *H. pylori*, achieving a detection range of 0.1–160 ng/mL and a limit of detection of 0.017 ng/mL, using graphitic carbon nitride to enhance signal sensitivity and stability. Roushani, Sarabaegi & Hosseini (2024) introduced a hybrid aptamer-molecularly imprinted polymer (MIP) on carbon nanocapsule-modified electrodes. This technique has improved selectivity, stability and cost-effectiveness. These platforms highlight the adaptability of aptamers in diverse sensing systems, offering potential for both clinical diagnostics and POC applications, with the added capability to differentiate *H. pylori* in complex biological samples.

Beyond diagnostic, aptamers have also been explored for therapeutic interventions against *H. pylori*. Xia et al. (2020) reported that aptamer HA6 targeting the HpaA adhesin effectively disrupts IL-21-mediated MMP-2 and MMP-9 expression in AGS cells, thereby inhibiting gastric cancer metastasis. Complementing this approach, Yuan et al. (2023) developed aptamers targeting the blood group antigen-binding adhesin (BabA) to block bacterial adhesion to the host cells. Flow cytometry, colony count assays, and mouse models demonstrated reduced bacterial colonisation and attenuated inflammatory responses, with decreased levels of tumour necrosis factor-α (TNF-α), IL-4, IL-6, and IL-8 in gastric mucosal tissue.

Collectively, these studies highlight aptamers’ dual diagnostic and therapeutic potential for the management of *H. pylori* infection. Diagnostic aptamers enable rapid, selective detection, while therapeutic aptamers show promise in disrupting pathogenic mechanisms. However, most reported systems remain preclinical; further validation in clinical samples and comprehensive *in vivo* studies are essential to advance aptamer-based strategies toward clinical translation.

### Leptospira

Leptospirosis is an emerging zoonosis caused by the Gram-negative *Leptospira* bacteria (Abd Rahman et al., 2021; Samrot et al., 2021). Pathogenic and intermediate *Leptospira* cause disease in humans, while saprophytic strains found in the environment do not (Benacer et al., 2013). Transmission typically occurs *via* direct contact with infected animals or contaminated environments, particularly in poor sanitation or during extreme weather events such as flooding (Abd Rahman et al., 2021). The WHO estimated over one million people are infected with 58,900 fatalities annually (Costa et al., 2015).

Conventional diagnostic methods, such as enzyme-linked immunosorbent assay (ELISA) and microscopic agglutination test (MAT), detect IgG and IgM produced by the human immune system after seven days of *leptospira* infection (Sakhaee et al., 2010). This will delay treatment and risk the development of Weil’s disease or even death. Therefore, neither method is suitable for diagnosing leptospirosis in its early infection stages. PCR can also be used to diagnose leptospirosis, but requires specialised equipment and expertise, limiting POC applications. The innovative aptasensor can be utilised to address the limitations.

Studies have highlighted the ability of aptamers to diagnose leptospirosis with high sensitivity, specificity and versatility. Saranya et al. (2024) introduced a DNAzyme-based colourimetric aptasensor to detect *Leptospira interrogans* in 20 environmental water samples, achieving a detection limit of 119 CFU/mL, a dissociation constant (K_d_) of 356.6 nM, and minimal cross-reactivity. The label-free design assay provided a rapid, cost-effective, and field-deployable tool for monitoring waterborne *Leptospira* contamination. Kositanont et al. (2023) developed an electrochemical aptasensor utilising the RNA aptamer ETFB3-63, targeting the Electron Transfer Flavoprotein Subunit Beta (ETFB), enabled direct detection in complex matrices such as serum without extraction steps and demonstrating a linear detection range of 50–500 nM.

Research targeting the conserved outer membrane protein *lipL32* has further advanced aptamer-based diagnostics. Shien Yeoh et al. (2023) employed a modified tripartite-hybrid SELEX approach to isolate RNA aptamers targeting *lipL32* with LepRapt-11, which exhibited excellent binding properties and diagnostic potential in an enzyme-linked aptasorbent assay (ELASA). In parallel, Vishwakarma, Meganathan & Ramya (2023) also developed a colorimetric aptamer-AuNP assay capable of detecting *Leptospira* in 25 water samples with a LOD of 57 CFU/mL supporting its application in environmental surveillance. Expanding on this work, Yeoh, Tang & Citartan (2023) further developed the first DNA aptamer and hybrid-heterodimeric aptamer, LepDapt-5a, against *lipL32*, integrating multiple diagnostic formats including direct ELASA, sandwich ELASA and aptamer-based dot assay. These platforms achieved detection limits within a clinically relevant range of leptospiremia, highlighting their potential for early-stage diagnosis.

Beyond diagnostics, aptamers have also been explored for therapeutic purposes. Hsu et al. (2024) generated aptamers specific to *lipL32* *via* SELEX and tested their ability to inhibit *Leptospira* invasion by disrupting the interaction between *lipL32* and ECM components. In a Syrian golden hamster model, treatment with the *lipL32*-targeting aptamer (L32AP) significantly reduced bacterial invasion and kidney colonisation. The treatment results show a substantial improvement in the survival rate, exceeding 50% compared to untreated controls. Although L32AP did not suppress *lipL32*-induced TLR2-mediated inflammatory signalling, these findings highlight the potential of aptamers as therapeutic tools for minimising tissue damage and disease progression (Hsu et al., 2024).

### Aptamers applications for viral zoonotic pathogens

Viral zoonoses are zoonotic diseases caused by viruses that spread to humans through animal bites, direct contact or contaminated food, water or the environment. In this manuscript, a few viral zoonoses, including monkeypox, rabies and ZIKA are discussed.

### Monkeypox virus

Mpox, previously known as Monkeypox, is a viral zoonosis identified in Africa but has spread to more than 120 countries, including Asia (Saied et al., 2022; World Health Organization (WHO), 2024a). It is caused by the oval-shaped Monkeypox virus (MPXV) from Orthopoxvirus genus (Alakunle et al., 2020). This virus can be found in rodents such as dormice, rats, monkeys and squirrels (Chandra Das et al., 2023). According to World Health Organization (WHO) (2024a), Mpox is transmitted through two routes: animals to humans or humans to humans. Infected animals can transmit the virus to humans through bites or scratches or through actions like hunting, skinning, trapping, handling carcasses, cooking, or eating animals while human transmission occurs through close contact with infected patients or animals, contaminated material such as mucosal lesions, bodily fluids, or wounds (World Health Organization (WHO), 2024a).

From January 2022 to August 2024, there have been more than 100,000 laboratory-confirmed cases with over 220 confirmed deaths reported by World Health Organization (WHO) (2024a). Mpox patients may present with symptoms such as rash, sore throat, fever, swollen lymph nodes, skin lesions, and muscle pain, which are very similar to those of smallpox disease (World Health Organization (WHO), 2024a). Therefore, it remains a challenge to diagnose using observation. Healthcare professionals perform molecular tests such as PCR, RT-PCR and whole-genome sequencing using the specimens obtained directly from rash, skin lesions or bodily fluids to detect Mpox. However, these methods require skilled personnel with access to expensive lab equipment (Islam et al., 2023). Antibody detection techniques may not be helpful because they can’t distinguish one *Orthopoxvirus* from another (World Health Organization (WHO), 2024a). This shows the importance of technology like aptamer to detect MPXV.

Studies on the therapeutic and diagnostic applications of aptamers for Mpox have been carried out. Recently, in 2024, Han and coworkers developed a CRISPR/Cas12a-mediated aptasensor targeting the A29 protein of MPXV. Utilising a pair of aptamers identified through SELEX, the aptasensor employs bivalent aptamer recognition to initiate proximity switch probes and cascade strand-displacement reactions, which trigger CRISPR/Cas12a DNA trans-cleavage. This innovative approach achieved an ultra-sensitive detection range of 1 ng/mL to 1 µg/mL, with a LOD of 0.28 ng/mL. Furthermore, the aptasensor maintained its efficacy after 6 months of storage and demonstrated over 96.9% recovery in spiked protein samples. This shows the robustness of this aptasensor and its translational potential.

Complementing this approach, Wang et al. (2024) introduced a one-pot detection platform combining fluorogenic Mango III RNA aptamers, recombinase-aided amplification (RAA), and the CRISPR-Cas13a system. The system enabled rapid, highly sensitive detection of MPXV with a LOD as low as four viral DNA copies within 40 min without nucleic acid purification. Clinical validation confirmed its reliability and suitability for POC testing, particularly in resource-limited settings. Collectively, these studies highlight the promise of aptamer-integrated CRISPR platforms as rapid, sensitive, and field-deployable tools for Mpox surveillance and outbreak control.

### Rabies virus

Rabies virus (RABV) is a fatal zoonotic disease caused by the *Lyssavirus* genus from Rhabdoviridae family. It is a neurotropic virus with a bullet-shaped structure with five major genes: N, G, P, M, and L (Robertson, Marano & Johnson, 2012). Rabies viruses can be detected in human biological fluids, including cerebrospinal fluid (CSF), saliva, urine, tears, and the nape (Lippi & Cervellin, 2021). Although rabies is preventable through vaccination, outbreaks continue to occur, particularly in unvaccinated free-roaming and wild dog populations (Thanapongtharm et al., 2021). Insufficient public health infrastructure and weak enforcement of dog vaccination policies further exacerbate rabies transmission (Salomão et al., 2017). Every year, approximately 59,000 rabies-related fatalities are reported by WHO, with the highest recorded in Asia and Africa (Jane Ling et al., 2023; World Health Organization (WHO), 2024b).

Dogs remain the primary reservoir and transmitters of human rabies, accounting for approximately 99% of the reported cases. Other mammals, such as bats, raccoons, skunks, foxes, and wolves, can serve as hosts (Jane Ling et al., 2023). Rabies virus is abundantly present in the saliva of infected animals and is transmitted to humans through bites or scratches, causing viral entry into subcutaneous tissues (Lippi & Cervellin, 2021). Following infection, the virus travels retrogradely to the central nervous system *via* fast axonal transport along peripheral nerves, eventually causing fatal encephalitis (Lippi & Cervellin, 2021).

Current rabies diagnostics include direct fluorescence antibody test (dFAT), the rapid fluorescent focus inhibition test (RFFIT), and RT-PCR. These diagnostic methods detect viral antigens, neutralising antibodies, or viral RNA in tissue or blood samples (Condori et al., 2020). While these methods are reliable, they require specialised equipment, skilled personnel, and laboratory infrastructure, therefore limiting their suitability for POC or field deployment. Moreover, delays in diagnosis can hinder the timely administration of post-exposure prophylaxis (PEP), substantially increasing mortality risk. Rapid and accurate rabies detection is therefore critical, not only to guide timely PEP administration but also to prevent unnecessary PEP use in non-exposed individuals, highlighting the need for alternative diagnostic technologies such as aptasensors.

To date, most advances in rabies-related aptamer research have focused on the therapeutic rather than diagnostic applications. Liang et al. (2013) developed single-stranded DNA aptamers, FO21 and FO24, targeting the rabies virus CVS-11 strain and evaluated their protective efficacy in mouse models. Mice treated with aptamers 24 h prior to viral challenge exhibited significantly improved survival rates, with PEG-FO24 achieving 87.5% survival compared to 40% for PEG-FO21 at lower doses. Importantly, treatment with PEG aptamers alone did not result in significant weight loss, indicating favourable biocompatibility. In a follow-up study, Liang et al. (2014) identified a refined aptamer GE54, that conferred partial protection (25–33% survival) at 48 h post-infection and effectively inhibited viral replication in baby hamster kidney-21 (BHK-21) cells, as confirmed by virus titre assay and qRT-PCR.

More recently, Ren et al. (2021) introduced an innovative therapeutic strategy using aptamer-functionalised RVG-Apt-PEG-Silica gold nanorods (AuNRs) for targeted photothermal therapy (PTT). The aptamer ensured specific binding to RABV glycoproteins, while RABV glycopeptide (RVG) facilitated efficient central nervous system (CNS) delivery. This platform achieved approximately a 100-fold reduction in viral load *in vitro*. It significantly decreased viral RNA levels in mouse brain tissue following photothermal therapy (PTT), resulting in a 60% survival rate with minimal neuroinflammation or apoptosis. Similarly, Scott & Nel (2021) explored the potential of aptamers as an alternative to rabies immunoglobulin (RIG) in post-exposure prophylaxis (PEP). Their study demonstrated that aptamer UPRET 2.03 reduced viral RNA levels by 61.3% at 2 h post-infection while a chimaera combining UPRET 2.03 with siRNA achieved a 36.5% reduction at 24 h post-infection, supporting the feasibility of aptamer-based therapeutic interventions for rabies.

### ZIKA virus

Zika virus (ZIKV) is a mosquito-borne pathogen closely related to other medically significant flaviviruses, including yellow fever (YFV) and dengue (DENV). ZIKV transmission occurs through both vector-borne routes, such as *Aedes aegypti* and *Aedes albopictus*, and non-vector transmission, including blood transfusion, vertical/congenital transmission, sexual transmission, and organ transplantation (Pergolizzi et al., 2021). ZIKV infections are often asymptomatic or mild (headache, fever, joint pain, and myalgia). However, severe complications can occur, including microcephaly, transverse myelitis, Guillain–Barré syndrome (GBS), and neuropathy and adverse pregnancy outcomes, like premature delivery, stillbirth, or foetal loss (Dahiya et al., 2023).

Accurate diagnosis of ZIKV remains challenging due to symptom overlap with other flaviviral infections and high incidence of asymptomatic cases. Current diagnostic methods include serological and molecular methods. Serological assays, such as enzyme immunoassays (EIAs), immunofluorescence assays (IFAs), and neutralisation assays, are typically applied to detect antibodies after 7 days of symptom onset. However, they are prone to cross-reactivity with other flaviviruses, leading to false-positive results (Bhardwaj et al., 2021). Molecular methods, particularly RT-PCR, can achieve high diagnostic accuracy during early infection but require specialised equipment and trained personnel, which limits their suitability for POC applications (Da Silva et al., 2019; Dahiya et al., 2023). Due to these limitations, aptamers have emerged as an important alternative tool for rapid and specific ZIKV detection.

Almeida et al. (2022) developed a high-affinity aptamer (ZIKV60) targeting the non-structural protein 1 (NS1) of ZIKV using capillary electrophoresis-based SELEX. ZIKV60 exhibited a dissociation constant (K_d_) of 2.28 ± 0.28 nM and demonstrated strong specificity for ZIKV NS1 over DENV and YFV NS1. When integrated into graphene field-effect transistor devices, the aptamer enabled ultrasensitive detection of the NS1 protein in human serum at concentrations as low as 0.01 pg/mL, despite the presence of blood components (Almeida et al., 2022). This platform highlights the potential of ZIKV60-based biosensors for rapid and specific differential diagnosis of the Zika virus.

Complementary advances have focused on rapid electrical biosensors for ZIKV detection, particularly in asymptomatic infections. Jang et al. (2023) developed an electrochemical biosensor employing a truncated DNA aptamer (T-ZV apt) immobilised on an interdigitated gold micro-gap electrode and enhanced by alternating current electrothermal flow (ACEF) to improve target capture efficiency. The T-ZV apt retained binding affinity comparable to the full-length sequence while reducing manufacturing costs. Using pulse-voltammetry, the biosensor detected the ZIKV envelope protein in diluted serum within 10 min, even achieving a LOD of 90.1 pM (Jang et al., 2023). Atomic force microscopy confirmed aptamer-target interactions, and sensor responses increased linearly with protein concentrations, supporting its potential for rapid and cost-effective POC testing.

In addition to electrochemical platforms, Nguyen et al. (2022) developed a peptide aptamer-based flow immunochromatographic test strip (FICT) for rapid detection of ZIKV. The test strip can distinguish ZIKV from DENV. Peptide aptamers targeting ZIKV envelope protein selected based on binding affinity and a B2.33-P6.1 peptide pair demonstrated optimal performance. The assay achieved an LOD of 2 × 10^4^ TCID_50_/mL and successfully discriminated ZIKV from DENV in human serum and urine samples, with consistent sensitivity and stability (Nguyen et al., 2022). These findings highlight the potential of peptide aptamer-based lateral flow systems, coupled with *in-silico* modelling, for rapid and effective ZIKV detection.

### Aptamer applications for parasitic zoonotic pathogens

Parasitic zoonoses are diseases transmitted from animals to humans caused by parasitic organisms such as protozoa, helminths, and ectoparasites. These parasites can be transmitted through many routes, including direct contact with infected animals, contaminated food or water, or vector-borne transmission by insects like mosquitoes. Some common examples of parasitic zoonoses such as malaria and toxoplasmosis, have complex life cycles which complicate diagnosis and treatment.

### *Plasmodium* spp.

*Plasmodium* spp. is a protozoan parasite that causes malaria, a life-threatening disease that occurs in tropical and subtropical regions (Islam, Dhar & Rahman, 2023). Five species of *Plasmodium* spp. consisting of *P. falciparum*, *P. malariae*, *P. ovale*, *P. vivax*, and *P. knowlesi* are known to infect humans, with *P. falciparum* causing the most severe disease (White, 2008; Islam, Dhar & Rahman, 2023). Malaria transmission occurs through the bite of an infected female *Anopheles* mosquito, after which the parasite undergoes hepatic and erythrocytic development, leading to clinical manifestations ranging from fever to severe organ complications (Msugupakulya et al., 2023; World Health Organization (WHO), 2022).

Conventional malaria diagnostics include Giemsa-stained microscopy, rapid diagnostic tests (RDTs), and nucleic acid amplification tests (NAATs). Although microscopy remains the gold standard, it is labour-intensive and operator-dependent. RDTs offer rapid detection but suffer from limited sensitivity at low parasitaemia and the inability to quantify parasite load. NAATs provide superior sensitivity but are costly and impractical in resource-limited settings (Fitri et al., 2022; Calderaro, Piccolo & Chezzi, 2024). These limitations highlight the need for alternative diagnostic strategies.

Aptamer-based biosensors have emerged as a powerful tool for malaria detection due to their high specificity, stability, low cost, and compatibility with POC platforms. Electrochemical aptasensors targeting Plasmodium lactate dehydrogenase (PfLDH) and histidine-rich protein 2 (PfHRP2) have demonstrated high sensitivity at clinically relevant parasitaemia levels. For example, Figueroa-Miranda et al. (2021) developed a flexible, disposable electrochemical aptasensor that detects *P. falciparum with* sensitivity exceeding the WHO standards. Similarly, Lo et al. (2021) designed a methylene blue labelled aptasensor targeting PfHRP2, which achieved a LOD of 3.73 nM and has a good stability in serum.

Advanced platforms such as graphene-based BioFET aptasensors by Figueroa-Miranda et al. (2022) further improved sensitivity, achieving femtomolar detection limits (0.78 fM) and wide dynamic ranges suitable for asymptomatic infection monitoring. Portable systems such as smartphone-based fibre-optic aptasensors targeting PfGDH developed by Sanjay et al. (2020) offered cost-effective, field-deployable diagnostic solutions, achieving a LOD of 264 pM.

Beyond diagnostics, aptamers have shown potential as therapeutic agents against malaria by targeting essential parasite mechanisms and host-parasite interactions. Pitolli et al. (2024) developed RNA aptamers targeting tRip, a membrane protein responsible for the import of exogenous transfer RNA (tRNA) into *Plasmodium spp*. These aptamers competitively inhibited tRip, significantly impairing parasite development, and contained a conserved five-nucleotide motif critical for target binding. In a complementary host-targeted approach, Nik Kamarudin et al. (2020) isolated RNA aptamers targeting human CD36, a receptor that mediates cytoadherence of *Plasmodium*-infected erythrocytes. The aptamers, RC60 and RC25 effectively blocked erythrocyte adhesion to vascular endothelium, suggesting their potential as adjunct therapies to reduce severe malaria complications (Nik Kamarudin et al., 2020).

#### Toxoplasma gondii

Toxoplasmosis, a parasitic infection caused by *Toxoplasma gondii*, which infects warm-blooded animals, including cats, humans and other mammals. Most human infections are acquired through the consumption of undercooked contaminated meat, exposure to oocysts in faecal matter or congenital transmission. The disease is typically mild or asymptomatic in immunocompromised patients, pregnant women, and foetuses, while healthy individuals may experience mild flu-like symptoms such as fever, lymphadenopathy, and muscle aches. However, toxoplasmosis can lead to serious conditions, including toxoplasmic encephalitis in patients with weakened immune systems, such as organ transplant patients or those living with HIV/AIDS (Dian, Ganiem & Ekawardhani, 2023).

Time and accuracy are important for managing asymptomatic or chronic *T. gondii* infections, as early detection can prevent neurological and systemic damage and the spread of the disease. Conventional diagnosis primarily relies on serological assays such as ELISA, that detects anti-*T. gondii* antibodies. However, these tests cannot reliably distinguish between active and past infections. Polymerase chain reaction (PCR) offers high specificity for parasite DNA detection but requires specialised equipment and trained personnel, which limits its use in POC settings. These diagnostic methods present challenges related to speed, cost, and detection efficiency. Aptamer-based detection methods have therefore emerged as promising tools for direct detection of *T. gondii* antigens.

Surface antigen 1 (SAG1) is a membrane-associated surface antigen that is expressed during the tachyzoite stage of *T. gondii* (Liu et al., 2022). It plays an important role in host cell adhesion and invasion, thus making it an attractive diagnostic target (Khodadadi et al., 2021; Qi et al., 2022; Liu et al., 2022). Cui et al. (2022) reported SELEX-derived DNA aptamers with strong binding affinity for the SAG1 protein of *T. gondii*. The authors used direct enzyme-linked aptamer assay (DELAA) to detect circulating SAG1 antigens in parasite lysate and sera of infected humans and animals. The DELAA assay demonstrated high sensitivity and specificity, outperforming a commercial ELISA kit with good repeatability, indicating its reliability for toxoplasmosis diagnosis.

In addition to SAG1, rhoptry protein 18 (ROP18) is a major virulence factor involved that modulates the host innate immune response and promoting parasite survival. Vargas-Montes et al. (2019) developed aptamers targeting the ROP18 protein using enzyme-linked aptamer assay (ELAA). This assay enabled the detection of both total antigens from the RH strain and the recombinant ROP18 protein. Notably, the ELAA test positive results showed a strong correlation with severe congenital toxoplasmosis in human blood samples. This suggested the potential of ROP18-targeting aptamers as clinically relevant biomarkers for disease severity.

Electrochemical aptasensor strategies have also been explored to enhance diagnostic performance. Luo et al. (2013) developed a dual-aptamer electrochemical biosensor for detecting *T. gondii* antibodies, in which signal changes occurred only when both aptamers bound the target antibody simultaneously. This dual-recognition method significantly improved the specificity and sensitivity compared to single-aptamer approaches. This highlights its potential utility in resource-limited settings and high-risk populations, including pregnant women and immunocompromised individuals.

### Emerging zoonotic threats: new horizons for aptamer technology

#### Paramyxoviruses: Nipah virus and Hendra virus

Paramyxoviruses, particularly Nipah and Hendra viruses, represent critical zoonotic threats with significant pandemic potential. These viruses cause severe respiratory and neurological disease in humans, with mortality rates ranging from 40–75% in Nipah virus outbreaks (Islam, Ibrahim & Hemo, 2024). Currently, no licensed vaccines or specific antiviral treatments are available for these pathogens, highlighting an urgent need for rapid diagnostic tools and therapeutic interventions (Chan et al., 2025). The lack of POC diagnostics hampers outbreak response efforts, while the absence of targeted therapeutics leaves clinicians with only supportive care options.

Aptamers present a promising approach to address these gaps, as they can be generated rapidly and tailored to bind specific viral fusion proteins and other virulence factors. The SELEX process can be completed within weeks, enabling rapid responses to emerging paramyxovirus threats. Aptamers could be designed to target Nipah virus receptor-binding F protein or the V protein involved in immune evasion, offering novel therapeutic avenues that complement conventional antiviral approaches (Chakraborty, Saha & Bhattacharya, 2024). Their chemical stability and ease of modification make them particularly suitable for developing both aptamer-based biosensors and therapeutic inhibitors against these highly pathogenic viruses.

Future research should prioritise the development of aptamers targeting conserved regions of paramyxoviral proteins to ensure broad-spectrum efficacy across different strains. Advanced aptamer-based biosensors integrated with microfluidic platforms should be explored to enable rapid field deployment for outbreak surveillance. In addition, chemical modifications that improve aptamer stability and *in vivo* delivery should be optimised to strengthen therapeutic utility. Collaborative efforts between aptamer researchers and paramyxovirus experts will be essential to accelerate translation from laboratory findings to clinical applications.

#### Filoviruses: Ebola virus and Marburg virus

Filoviruses, including Ebola virus (EBOV) and Marburg virus (MARV), cause severe hemorrhagic fevers with mortality rates often exceeding 50% (Rougeron et al., 2015). These viruses pose significant global health security threats, as demonstrated by the West African Ebola outbreak in 2014–2016 (Ravi, Snyder & Rivers, 2019). Current diagnostic methods often require specialised laboratory facilities and trained personnel, limiting their use in the resource-limited settings where outbreaks frequently occur. While some therapeutic options (*e.g*., vaccines and monoclonal antibodies for Ebola) are now available, the need for rapid POC diagnostics and additional therapeutic modalities remains critical for effective outbreak response.

Groundbreaking research has identified RNA aptamers targeting the Ebola virus VP35 protein, a multifunctional factor crucial for viral replication and immune evasion (Binning et al., 2013). These aptamers bind distinct regions of the VP35 interferon inhibitory domain with high affinity (10–50 nM) and can disrupt key protein–protein interactions. Notably, the aptamers compete with double-stranded RNA for VP35 binding and inhibit the function of viral polymerase complex, demonstrating therapeutic potential (Binning et al., 2013). Computational studies have further elucidated binding sites and mechanisms of VP35 inhibition, supporting the development of aptamer-based countermeasures against filoviruses. Priority should be given to expanding aptamer development beyond VP35 to target other essential filoviral proteins such as VP40 and the viral glycoproteins.

Field-deployable aptamer-based diagnostic platforms should be developed and validated in endemic regions while chemical modifications to improve aptamer stability under tropical conditions warrant further investigation. In parallel, integrating aptamer therapeutics with existing treatment protocols (for example, alongside antiviral antibodies) requires systematic evaluation through preclinical and clinical studies.

#### Neglected bacterial zoonoses: *Brucella* spp. and *Yersinia pestis*

Brucellosis affects over 500,000 people worldwide each year, with underdiagnosis remaining a major problem due to non-specific symptoms and limited diagnostic capacity in endemic regions (Ahangari et al., 2024). Plague, caused by *Yersinia pestis*, remains a threat with approximately 1,000–3,000 cases reported each year globally (Shafiei et al., 2024). Current diagnostic methods for both diseases rely on time-consuming culture-based techniques, require specialised facilities, and pose safety risks to laboratory personnel. Rapid, sensitive diagnostic tools suitable for resource-limited settings are urgently needed for effective disease management and outbreak control (Ahangari et al., 2024).

Recent research has reported the development of a gold nanoparticle-based aptamer colourimetric sensor for *Brucella* detection with high sensitivity, achieving a LOD as low as 15 CFU/mL (Ahangari et al., 2024). This performance can surpass conventional PCR sensitivity while providing rapid results suitable for field deployment. For plague surveillance, DNA aptamers have been selected against the *Y. pestis* F1 antigen, a key capsule protein used for plague diagnosis (Shafiei et al., 2024). These aptamers show promise for diagnostic applications and environmental surveillance of plague reservoirs.

Validation of aptamer-based Brucella sensors in clinical samples and field conditions should be prioritised to support translation to POC use. The development of multiplex aptamer assays capable of distinguishing among *Brucella* species could improve diagnostic specificity. For plague detection, aptamer-based sensors for environmental monitoring of *Y. pestis* in flea and rodent populations should be developed. Integration of these aptamer platforms with mobile health technologies could enhance surveillance capabilities in remote endemic areas.

#### Protozoan parasites beyond *Plasmodium*: *Leishmania* and *Trypanosoma*

Neglected tropical diseases caused by protozoan parasites affect over one billion people worldwide, with leishmaniasis causing 20,000–30,000 deaths annually and Chagas disease affecting 6–7 million people globally (Janegitz, 2024). Current diagnostics often lack sensitivity, particularly in early-stage infections, and rely on specialised laboratory infrastructure consistently unavailable in endemic regions (Janegitz, 2024). The absence of rapid, POC diagnostic tools significantly hampers disease management and control efforts, while therapeutic monitoring remains challenging due to limited diagnostic options (Papi et al., 2025).

Electrochemical aptamer-based sensors show promise for the detection of *Leishmania*, although research remains limited (Janegitz, 2024). A qualitative electrochemical paper-based analytical device (e-PAD) using unmodified screen-printed electrodes and antibody-based recognition for *Leishmania* detection demonstrates the feasibility of adapting POC platforms through aptamer-based systems (Papi et al., 2025). For *Trypanosoma* species, aptamer research remains at an early stage, with most studies focusing on proof-of-concept development rather than clinical validation (Nagarkatti et al., 2012). However, the stability and specificity of aptamers make them particularly suitable to address the diagnostic gaps in these neglected diseases.

Further effort should prioritise systematic aptamer selection targeting key *Leishmania, T. cruzi*, and *T. brucei* surface proteins and secreted factors. The development of multiplexed aptamer platforms capable of distinguishing between species and disease stages could significantly improve diagnostic capability (Gállego, Schijman & Alonso-Padilla, 2020). Field-deployable aptamer biosensors for resource-limited settings require development and validation, alongside exploration of aptamer-based therapeutic applications, such as drug delivery and direct antiparasitic effects, to provide novel treatment options.

### Next-generation directions: from lab to field and beyond

#### Theranostic aptamers

Aptamers’ unique physicochemical properties make them ideal for theranostic applications, serving as both diagnostic tools and therapeutic agents within the same molecular framework. This dual functionality boost cost-effectiveness and efficiency in zoonotic disease outbreak surveillance and control.

Examples from this review demonstrate the theranostic potential (Table 3). For instance, *H. pylori* aptamers targeting CagA and BabA virulence factors have also been reported to inhibit the bacterial adhesion and colonisation (Yadav et al., 2023; Yuan et al., 2023; Roushani, Sarabaegi & Hosseini, 2024). Similarly, aptamers targeting the *lipL32* protein of *Leptospira* also interfere with bacterial invasion and interactions with the extracellular matrix (ECM) (Yeoh, Tang & Citartan, 2023; Hsu et al., 2024).

**Table 3:** List of aptamers that have the function of theranostics.

Pathogen	Diagnostic target	Therapeutic mechanism	Aptamer name	References
*H. pylori*	CagA, HpaA, BabA	Inhibits adhesion; reduces inflammation	Hp4, HA6, A10/A41/A42	Yan et al. (2019), Xia et al. (2020), Yuan et al. (2023)
*Leptospira*	*LipL32*, ETFB	Blocks ECM interaction	LepRapt-11, L32AP	Hsu et al. (2024), Yeoh, Tang & Citartan (2023)
*ZIKA*	NS1 protein	Potential to block viral entry	ZIKV60	Almeida et al. (2022)

Integrating aptamers into multifunctional nanosystems (*e.g*., aptamer-nanoparticle conjugates) may further enhance their theranostic value *via* simultaneous imaging, pathogen neutralisation, and targeted delivery. Future research should prioritise developing modular aptamer designs for rapid adaptation to emerging pathogens, supporting both diagnostic precision and therapeutic impact.

#### Artificial intelligence for aptamer selection

Advances in artificial intelligence (AI) and machine learning accelerate identification and characterisation of aptamers with high specificity towards pathogens. *In-silico* approaches can improve the SELEX workflow and support aptamer design, structure prediction and binding analysis (Buglak et al., 2020). The key steps in computational aptamer modelling include secondary and tertiary structure prediction, molecular docking and molecular dynamics simulation (Buglak et al., 2020). Several platforms with different computational algorithms have been developed to support these stages (Chen et al., 2021). Studies by Almazar, Mendoza & Rivera (2023) and Azlina et al. (2023), reported aptamers targeting *Leptospira* and SARS-CoV-2 using *in-silico* methods. Azlina et al. (2023) further demonstrated that the M49 DNA aptamer can also bind the structurally similar ORF7a protein of SARS-CoV-2, suggesting that protein structural similarity may enable cross-targeting by the same aptamer.

Machine learning (ML) approaches can be broadly categorised into similarity-based or feature-based (Chen et al., 2021). Similarity-based ML uses sequence-based and structure-based clustering tools. Sequence-based tools enable rapid analysis of large SELEX datasets by identifying similar aptamer sequences. However, limited structural information can compromise binding affinity predictions. In contrast, structure-based clustering tools evaluate binding potential based on specific structural motifs offering more reliable predictions at the cost of longer processing times. Examples of structure-based models include AptaTrace and APTANI (Chen et al., 2021). Feature-based ML approaches aim to predict outcomes for unlabelled data by learning patterns from labelled training datasets. While promising, these models depend heavily on the quality of training data and may struggle to reliably capture key features, leading to uncertain predictions.

#### Microfluidics

The ultimate goal of aptamer technology is POC adaptability. Microfluidic systems comprising micropumps, microchannels, microvalves and detection units have gained increasing attention for diagnosis and surveillance of microbial outbreaks (Zhao et al., 2024). These platforms typically require only a small volume of analytes (Xu, Yang & Wang, 2010; Khan & Song, 2020). Within microfluidic chips cassettes, sample pretreatment, micro-mixing, micro-reaction and signal readout can be integrated into a single miniaturised workflow (Zhao et al., 2024). In recent years, aptamer-based microfluidics enabling real-time detection has been developed to detect zoonoses, exosomes, plant diseases and foodborne bacterial pathogens (Hao et al., 2019; Verma, Kulkarni & Pandya, 2022; Zhao et al., 2024; Hu & Gao, 2025; Domingues et al., 2025).

The combination of microfluidics with electrochemical detection provides robust, quantitative results suitable for field deployment (Watkins et al., 2023). Recent innovations include paper-based microfluidic devices that eliminate the need for external pumps and complex fluidic control systems (Mishra et al., 2022). A paper-based microfluidic aptasensor has demonstrated detection of the bacterial pathogen *Listeria monocytogenes* with high specificity despite the presence of non-target bacteria such as *Bacillus subtilis* and *Escherichia coli* (Mishra et al., 2022).

Microfluidic aptamer sensors have shown particular promise for continuous monitoring, with some systems demonstrating week-long operation in complex biological fluids (Watkins et al., 2023). Advanced surface chemistry modifications enable stable aptamer immobilisation and reduced biofouling, critical factors for long-term sensor performance (Watkins et al., 2023). Future developments should focus on standardised microfluidic platforms that can accommodate diverse aptamer assays while maintaining consistent performance. Integration with wireless communication technologies could enable real-time data transmission for surveillance and outbreak response applications.

### Critical gaps hindering aptamer translation

#### Lack of clinical translation and approved aptamer drugs

Despite over two decades of intensive aptamer research, clinical translation remains limited, with only pegaptanib approved by the FDA for the treatment of macular degeneration. To date, aptamer therapeutics targeting zoonotic diseases have not advanced to clinical trials, highlighting a critical gap between laboratory innovation and practical application. This translational bottleneck stems from multiple factors, including regulatory unfamiliarity with aptamer-based therapeutics, challenges in demonstrating clinical efficacy, and difficulties in scaling manufacturing processes. The complex regulatory landscape for nucleic acid therapeutics creates additional barriers, as existing frameworks were primarily designed for small-molecule drugs and biologics.

Proposed solutions include establishing specialised regulatory pathways for aptamer-based therapeutics and developing standardised protocols for clinical evaluation and testing. Enhanced collaboration among academic researchers, pharmaceutical companies, and regulatory agencies could accelerate the development of appropriate guidelines and approval processes.

#### Underrepresentation of certain pathogen classes

Current aptamer research shows significant bias toward well-studied pathogens like HIV and SARS-CoV-2, while neglecting important zoonotic threats (Janegitz, 2024). Despite affecting over one billion people globally, parasitic diseases have received minimal attention in aptamer research, with no approved aptamer therapeutics for any parasitic infection (Janegitz, 2024). Fungal zoonoses, rickettsial diseases, and numerous viral zoonoses remain severely underexplored in aptamer applications (Janegitz, 2024). This research gap reflects funding priorities and technical challenges rather than clinical need or potential impact (Aljazzar, 2025).

Systematic research initiatives targeting neglected zoonotic pathogens should be established, with dedicated funding streams for aptamer development against underrepresented pathogen classes (Janegitz, 2024). International collaborative networks could coordinate research efforts and share resources to address these gaps more effectively (Aljazzar, 2025).

#### Challenges with stability, delivery, and real-world deployment

Nuclease degradation and short half-life in biological fluids represent major obstacles limiting aptamer therapeutic applications. Most aptamer-based diagnostics remain at prototype stages without field validation in complex, resource-limited environments where zoonotic diseases are most prevalent. The gap between laboratory performance and real-world effectiveness reflects insufficient testing under realistic conditions, including temperature extremes, humidity, and sample matrix complexity (Liu & Panagiotakos, 2022). Current chemical modification strategies to improve stability can compromise binding affinity or increase manufacturing costs.

Advanced delivery systems incorporating nanoparticle carriers and chemical modifications should also be developed to address stability challenges while maintaining therapeutic efficacy (Abrishami et al., 2024). Field validation studies in endemic regions are essential to demonstrate real-world performance and identify practical deployment challenges.

#### Regulatory and standardisation gaps

The lack of standardised protocols for aptamer validation and regulatory approval creates significant barriers to clinical translation. Unlike antibodies, which benefit from well-established validation frameworks, aptamers lack consensus guidelines for performance evaluation, quality control, and clinical assessment. This regulatory uncertainty discourages investment and slows development timelines for commercial applications. The need for specialised expertise within regulatory agencies to evaluate aptamer-based products further complicates the approval process (Syed et al., 2024).

The development of international standards for aptamer characterisation, validation, and clinical evaluation should be prioritised through collaboration among regulatory agencies, scientific societies, and industry stakeholders. The establishment of reference materials and standardised assays would facilitate regulatory review and improve reproducibility across different research groups (Syed et al., 2024).

## Conclusion

Diagnosing zoonotic diseases remains challenging due to their overlapping clinical symptoms and delayed diagnosis, which leads to increased morbidity and mortality. In parallel, the rise of antimicrobial resistance also limits therapeutic options for bacterial zoonoses, highlighting the need for alternative approaches for effective detection and management. Aptamers, short, single-stranded oligonucleotides, have emerged as promising tools for diagnostics and therapeutics because of their high specificity and sensitivity. As diagnostic recognition elements, aptamers offer advantages over conventional antibodies, including improved stability, lower production costs, and ease of chemical modification. These features have enabled the development of diverse aptamer-based biosensing platforms with promising analytical performance. However, most remain at the proof-of-concept or preclinical stage, with limited validation in clinically relevant samples. Beyond diagnostics, aptamers also show therapeutic potential by neutralising virulence factors and modulating host-pathogen interactions, although challenges related to *in vivo* stability, delivery, and regulatory approval persist.

Overall, aptamer-based technologies represent a promising yet evolving approach to addressing zoonotic diseases. Future research should prioritise improving robustness in complex biological matrices, expanding clinical validation, and integrating emerging technologies such as microfluidics and artificial intelligence-assisted design to facilitate translation. Addressing these barriers will be essential for realising the full potential of aptamer-based diagnostics and therapeutics in zoonotic disease control.

## Additional Information and Declarations

### Data Availability

The following information was supplied regarding data availability:

This is a literature review.
